# Editorial: Towards a basic standard methodology for international research in psychology

**DOI:** 10.3389/fpsyg.2023.1170108

**Published:** 2023-03-24

**Authors:** Milagros Ocalin Sánchez Hernández, Fco. Pablo Holgado-Tello, Miguel Angel Carrasco, Salvador Chacón-Moscoso, Susana Sanduvete-Chaves, José Antonio Lozano-Lozano

**Affiliations:** ^1^Department of Psychology, National Autonomous University of Nicaragua at León (UNAN-León), León, Nicaragua; ^2^Department of Methodology of Behavioral Science, National University of Distance Education (UNED), Madrid, Spain; ^3^Department of Psychology of Personality, Assessment and Treatment, National University of Distance Education (UNED), Madrid, Spain; ^4^Department of Experimental Psychology, Universidad de Sevilla, Seville, Spain; ^5^Department of Psychology, Universidad Autónoma de Chile, Santiago, Chile; ^6^Institute of Biomedical Sciences, Universidad Autónoma de Chile, Santiago, Chile

**Keywords:** International Psychology, psychological research, methodology, data analysis, research designs, measurement

There is a need to promote the creation of referential sources to help the improvement of global scientific practice. This could answer new challenges such as open science development and the application of research focused on diversity and inclusiveness. Consequently, leading to international psychological research that ensures the methods applied in analysis across countries and cultural groups are reliable and valid.

In this Research Topic, we address the role of methodology in International Psychology through the compilation of theoretical and empirical articles including original research and systematic reviews that explore psychological phenomena with a global focus, and from different areas of Psychology. This encompasses Clinical, Developmental, Educational, Cross cultural, Sport, and Work and Organizational Psychology.

We consider these articles are contemporary and innovational because the authors highlight and apply methodological issues related to the measurement of psychological phenomena, research designs, data collection techniques, and data analysis techniques across different countries.

Among the concrete contributions to this Research Topic, there are two articles that explore symptoms and the diagnostic criteria of psychological disorders. In Spain, findings on the early detection and prevention of psychosis are highlighted in Ceballos-Munuera et al.. They found a jointly partial mediation of Aberrant Salient and Disorganized Dimension between Ideas of Reference and the Psychotic Dimension across a continuum from general population to clinically diagnosed patients. Evidencing that a set of vulnerabilities (unusual thought content) could lead to a high-risk general pathological state and proneness to psychosis in particular. In Chile, Fioravante et al. found statistically significant differences between groups of school-age children with an Attention Deficit Hyperactivity Disorder (ADHD) diagnosis and without ADHD. This result indicated the importance of appropriate criteria and procedures to establish a diagnosis and implement effective interventions.

Regarding the effect of contextual events throughout life and mental health, there are some studies focusing on the changes and the experience of COVID pandemic. Díaz-García et al. found there was a variation in lifestyles of Spanish elders, but despite knowledge about the clinical aspects of SARS-CoV-2 and the application of recommended preventive measures, authors found certain relationships remained unchanged such as the one with family and friends. Adding to this, Carr et al. explore whether or not the COVID-19 pandemic was impacting pivotal links between living wages and employee attitudes and subjective wellbeing in twin cohorts of low-waged workers across New Zealand. They found the need of considering subjective wellbeing in the context of a crisis for employee livelihoods and lives.

To explore Development and Educational Psychology phenomena, some authors used a more theoretical approach with the use of Systematic Reviews Research. Alarcón-Espinoza et al. identify for research on emotional self-regulation in childhood and adolescence that most authors apply mixed methodologies and there are problems related to the recording of the duration and sequence of behaviors, highlighting the use of guidelines as guides for future research. While Tronchoni et al. try to highlight pedagogical concerns related to the use of reading in university teaching. Through this work, the authors verified the lack of research on proposals regarding how this pedagogical strategy evolves for a more interactive learning practice.

Furthermore, some studies focus more on theoretical-methodological operationalization of psychological phenomena. Smith et al. explore the process of what people from different cultures do when they faced with complex problems by presenting two computer-simulated dynamic problems in which they were required to act quickly or cautiously. Participants were asked to think aloud in their native language while working on the tasks and the protocols were digitally recorded, transcribed, and coded by coders from each country in terms of the steps involved in complex problem solving and dynamic decision making. Only those protocols with more than 15 transitions were analyzed. Results highlight the importance of process analyses in different tasks and show how cultural background guides people's decisions under uncertainty.

Mohamed et al. study the application of Taguchi algorithm (applied mostly in engineering) for the analysis of relationships of depression and obesity indices with other indicators as socioeconomic, screen time, sleep time, fitness use, and nutrition. Results found that, although Taguchi method can estimates correlations even undetected by applying other common statistics techniques with psychological data, scholars need to be cautious with statistical methods for measuring and estimating their research variables.

García-Franco et al. present a method for the study and assessment of personality disorders, especially those of the dramatic and emotional type. It consists in the application of a Bayesian network whose parameters have been obtained by the Delphi method of consensus from a group of experts. The result is a probabilistic graphical model that represents the psychological variables related to the personality disorders together with their relations and conditional probabilities, which allow identifying the symptoms with the highest diagnostic potential. They discuss the need to validate the model in the clinical population along with its strengths and limitations.

Complementing previous studies, Arias-Pujol et al. analyze the psychotherapist-patient interaction in psychoanalytic psychotherapy in a single case of a 4-year-old boy with a diagnosis of severe autism spectrum disorder. They used an *ad hoc* observation instrument combining a field format and a category system. They also estimated the results by applying a polar coordinate analysis that allows to obtain an inter-relational map of the connections detected between the established focal behavior and the different categories. Particularly, the result of this study show which therapist behaviors are most useful for promoting social interaction in a child with severe autism.

Regarding the Psychometric contributions, Lee et al. validate the Korean version of the Student-Athletes' Motivation toward Sports and Academics Questionnaire (SAMSAQ) using exploratory structural equation modeling (ESEM). The results of this study illustrated that the SAMSAQ-KR appears to be a robust and reliable instrument.

In line with it, some articles demonstrate the application of new psychometric methods. Ünlü studies self-determination theory (SDT) by applying sets and relations instead of a common numerical approach. The applied technique is an inductive item tree analysis, which is an established method of Boolean analysis of questionnaires. The underlying models were computed within each of the intrinsic, identified, introjected, and external regulations, in autonomous and controlled motivations, and the entire motivation domain. In future studies, the approach of this article could be employed to develop adaptive assessment and training procedures in SDT contexts.

Liang et al. proposed a missing data model for not-reached items in cognitive diagnosis assessments. They simulated the model by estimating model parameters using Bayesian Markov chain Monte Carlo method. The model improved diagnostic feedback results and produced accurate item parameters when the missing data mechanism was non-ignorable. They also study the applicability of the model using a dataset from the 2018 Program for International Student Assessment computer-based mathematics cognitive test.

Cui et al. propose a stochastic approximation expectation maximization (SAEM) algorithm to estimate a model in which Ramsay-curve item response theory is incorporated in a three-parameter normal ogive (RC-3PNO) with non-normal latent trait distributions. They based this proposal considering that in real testing the assumption of the normality of latent traits in the estimation of item response models may be untenable. The simulation studies reveal that the SAEM algorithm produces more accurate item parameters for the RC-3PNO model than for a conventional one (3PNO), especially when the latent density is not normal, as in the cases of a skewed or bimodal distribution. The authors also demonstrate the application of the proposed algorithm using a PISA 2018 test dataset.

Finally, we would like to emphasize that the editors greatly appreciate the contributions received from the authors, which were compiled in this Research Topic. We also hope it would be of interest to readers and a source of ideas and motivation for the development of future work in Methodology for International Research in Psychology.

## Author contributions

MS, FH-T, and MC took the lead in writing the manuscript. SS-C, SC-M, and JL-L provided critical feedback and helped shape content of the manuscript. All authors reviewed the final version of the manuscript. All authors contributed to the article and approved the submitted version.

